# In Vitro Activities of Enantiopure and Racemic 1′-Acetoxychavicol Acetate against Clinical Isolates of *Mycobacterium tuberculosis*

**DOI:** 10.3390/scipharm85030032

**Published:** 2017-09-18

**Authors:** Saradee Warit, Kamolchanok Rukseree, Therdsak Prammananan, Poonpilas Hongmanee, Pamaree Billamas, Sarinya Jaitrong, Angkana Chaiprasert, Birgit U. Jaki, Guido F. Pauli, Scott G. Franzblau, Prasit Palittapongarnpim

**Affiliations:** 1 Tuberculosis Research Laboratory, Medical Molecular Biology Research Unit, BIOTEC, National Science and Technology Development Agency, Thailand Science Park, Pathum Thani 12120, Thailand; Saradee@biotec.or.th (S.W.); Therdsak@biotec.or.th (T.P.); Pamaree@biotec.or.th (P.B.); Sarinya.Jaitrong@biotec.or.th (S.J.); 2 Science and Liberal Art, Amnatcharoen Campus, Mahidol University, Bangkok 73170, Thailand; kamolchanok.ruk@mahidol.ac.th; 3 Department of Pathology, Faculty of Medicine Ramathibodi Hospital, Mahidol University, Bangkok 73170, Thailand; poonpilas.hon@mahidol.ac.th; 4 Department of Microbiology, Faculty of Medicine Siriraj Hospital, Mahidol University, Bangkok 73170, Thailand; Angkana.cha@mahidol.ac.th; 5 Institute for Tuberculosis Research, College of Pharmacy, University of Illinois at Chicago, Chicago, IL 60607, USA; bjaki@uic.edu (B.U.J.); gfp@uic.edu (G.F.P.); sgf@uic.edu (S.G.F.); 6 Department of Medicinal Chemistry and Pharmacognosy, College of Pharmacy, University of Illinois at Chicago, Chicago, IL 60607, USA; 7 Department of Microbiology, Faculty of Science, Mahidol University, Bangkok 73170, Thailand

**Keywords:** *Alpinia galanga*, 1’-*S*-acetoxychavicol acetate, anti-tuberculosis, drug resistance

## Abstract

In the process of evaluating the effect of several plant extracts against *Mycobacterium tuberculosis* using the Microplate Alamar Blue Assay (MABA), an extract of Thai herb *Alpinia galanga* rhizome and its major component, 1′-acetoxychavicol acetate (ACA), exhibited marked anti-tuberculosis activity. The minimal inhibition concentrations (MICs) of the *S*-enantiomer of ACA (*S*-ACA) against *M. tuberculosis* H37R*a* ATCC 25177 and H37R*v* ATCC 27294 strains were 0.2 µg/mL and 0.7 µg/mL, respectively. More than 95% of 100 drug-sensitive and 50 drug-resistant mycobacterial clinical isolates were inhibited by extracted *S*-ACA at 1.0 µg/mL. All of the remaining isolates were inhibited at 2.0 µg/mL. In contrast to the *S*-enantiomer, synthetic racemic 1′-*R,S*-ACA (*rac*-ACA) showed MICs of 0.5 µg/mL and 2.7 µg/mL for *M. tuberculosis* H37R*a* ATCC 25177 and H37R*v* ATCC 27294, respectively, suggesting that the anti-tuberculosis effect might be primarily due to the *S*-form. These observations were in line with the MICs of *rac*-ACA against 98% of 93 drug-resistant clinical isolates, which showed the effective inhibitory dose at 2.0 µg/mL. After exposure to 2.7 µg/mL of *rac*-ACA for at least 3 h, the tubercle bacilli were completely killed. These demonstrated that ACA had potent anti-TB activity.

## 1. Introduction

Tuberculosis (TB) is caused by *Mycobacterium tuberculosis* and constitutes a major health problem exacerbated by the global human immunodeficiency virus (HIV) pandemic and the emergence of drug-resistant strains, such as multidrug-resistant TB (MDR-TB) and extensively drug-resistant TB (XDR-TB) [1]. Consequently, novel anti-tuberculosis drugs are widely sought after entities.

Our laboratory has screened numerous medicinal plant extracts against the avirulent *M. tuberculosis* H37R*a* strain. The activity of 1′-*S*-acetoxychavicol acetate (*S*-ACA), a major secondary metabolite of a popular Thai herb, *Alpinia galanga* (Linn) Swartz (*A. galanga* (syn. *Languas galanga* (Linn) Stuntx), commonly known as greater galangal, gave the most promising primary screening result, with an activity comparable to that of known first-line drugs [2]. *A. galanga* belongs to the family Zingiberaceae and is commonly used in the preparation of Thai condiments and ethnomedicine. The crude rhizome extract of *A. galanga* has been shown to inhibit the in vitro growth of the avirulent *M. tuberculosis* H37R*a* strain [3], streptomycin-resistant *M. tuberculosis*, *M. avium* and *M*. *bovis* [4]. Soundhari and Rajarajan [5] reported a minimum inhibitory concentration (MIC) of 250 μg/mL of the lyophilized ethanolic extract of galangal against isoniazid-resistant isolates. An ethanolic extract at 50–100 μg/mL inhibited *M. tuberculosis* under axenic aerobic and anaerobic conditions and in an intracellular environment [6]. This is consistent with a previous report that *A. galanga* extracts are able to penetrate into human adenocarcinomic alveolar basal epithelial cells (A549) and act on the bacterium residing intracellularly [7]. Additionally, broad spectrum activities of *A. galanga* rhizome extracts toward other pathogens have been described since 1976. For instance, it was found that the essential oil of *A. galanga* contains ACA at a high level and could inhibit the growth of many dermatophytes that cause tinea or dermatophytosis [8]. Later, *A. galanga* crude extracts were found to have broad-spectrum activity against several human bacteria (e.g., *Bacillus subtilis* MTCC2391, *Enterobacter aerogenes*, *Enterobacter cloacae*, *Enterococcus faecalis*, *Escherichia coli* MTCC 1563, *Klebsiella pneumoniae*, *Pseudomonas aeruginosa* MTCC 6642, *Salmonella typhimurium*, *Staphylococcus aureus* and *Streptococcus epidermis*), with little toxicity to host cells [9].

Regarding the broad spectrum of its inhibitory activity, galangal rhizomes were extracted and fractionated with several polar and non-polar solvents including hexane, chloroform, ethanol, methanol and water to identify their components. Analysis by GC-MS and ^1^H-NMR, ^13^C-NMR found ACA as the major constituent (76%) together with minor components such as *p*-coumaryl diacetate (8.0%), 1’-acetoxyeugenol acetate (3.1%), palmitic acid (3.2%), 9-octadecenoic acid (2.3%), 1’-hydroxychavicol acetate, *p*-acetoxycinnamic alcohol, eugenol, β-bisabolene, β-farnesene and sesquiphellandrene [2,10,11,12].

**Figure 1:**
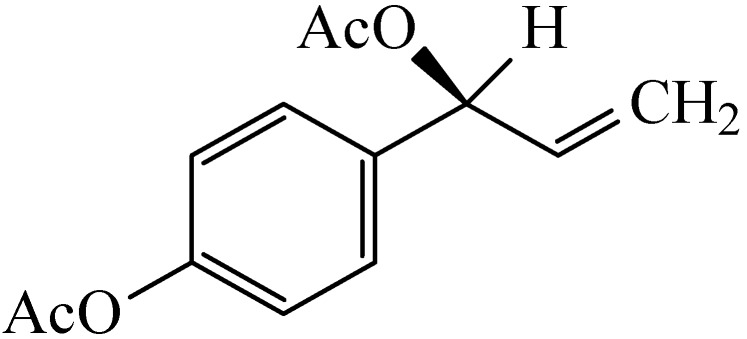
The chemical structure of 1′-*S*-acetoxychavicol acetate (*S*-ACA) from *A. galanga.*

Subsequently, ACA was identified as the component with the most promising anti-mycobacterial activity [2], and 30 mg of enantiopure ACA were isolated from 1 kg of dried *A. galangal* rhizome. Its structure was determined to be the *S*-enantiomeric form (Figure 1). In contrast, commercially sourced synthetic ACA was found to be racemic [13].

Mechanistic studies have shown that ACA causes bacterial membrane damage and cytoplasm coagulation in *Staphylococcus aureus*, leading to cell death [11]. In addition, ACA exhibited a blocking effect against human immunodeficiency virus type 1 replication at the stage of *Rev* transport and was found to act synergistically with other anti-HIV drugs [14]. However, the molecular target and mechanism of action of ACA are still ambiguous, and studies are limited mostly to eukaryotic cells [15]. Its target and mechanism of action in mycobacterial species have not been demonstrated yet.

The present study aimed to determine and compare the inhibitory activities against drug-sensitive and -resistant *M. tuberculosis* clinical isolates of the purified ACA (enantiopure *S*-form, *S*-ACA), as well as the synthetic form (racemic, *R*,*S* form, *rac*-ACA).

## 2. Materials and Methods

### 2.1. Bacterial Strains and Culture Condition

*M. tuberculosis* H37R*a* ATCC 25177 and H37R*v* ATCC 27294 including clinical isolates were grown in Middlebrook 7H9 medium (Becton Dickinson, Sparks, MD, USA) supplemented with 10% Oleic Acid-Albumin-Dextrose-Catalase (OADC) and 0.05% Tween-80 and on solid agar medium Middlebrook 7H10 medium (Becton Dickinson) supplemented with 10% OADC. One hundred fifty clinical isolates were collected from patients at Ramathibodi Hospital before 2002, and the other 93 isolates were obtained from the Drug-Resistant Tuberculosis Research Laboratory, Faculty of Medicine, Siriraj Hospital, Mahidol University, Bangkok, Thailand, between December 2003 and March 2013. The isolates were handled in a Level 3 biosafety laboratory.

### 2.2. Extraction and Purification of ACA

*S*-ACA was purified from the rhizome of *Alpinia galanga* (Linn) Swartz (*A. galanga*) as described previously [13]. Briefly, fresh rhizomes of *Alpinia galanga* (Linn) Swartz or *Languas galanga* (Linn) Stuntx were washed, sliced, dried at room temperature and ground into powder. One kilogram of the resulting powder was extracted with 3 L of dichloromethane for 72 h and filtered. The filtrate was concentrated by evaporation, applied to a silica gel column, eluted and fractioned with a step gradient of hexane:dichloromethane:methanol. After thin layer chromatography (TLC) and nuclear magnetic resonance (NMR) analysis, the major component, *S*-ACA, was identified and collected.

### 2.3. Minimum Inhibitory Concentration Determination Using Microplate Alamar Blue Assay

Rifampicin (RMP), isoniazid (INH), ethambutol (EMB), streptomycin (SM), ofloxacin (OFX) and kanamycin (KAN) were purchased from Sigma. Racemic *R*,*S*-1′-acetoxychavicol acetate (*rac*-ACA) was purchased from LKT laboratories (St. Paul, MN, USA; Product Number A0817). The MICs were determined as described previously [16]. Briefly, all mycobacterial strains were prepared from scoops of solid culture and suspended in PBS plus Tween-80, to reach a concentration equivalent to McFarland Standard No. 1 (approximately 3 × 10^7^ Colony Forming Units (CFU)/mL) and then diluted by Middlebrook 7H9 medium supplemented with 10% OADC to about 10^5^ CFU/mL. One hundred microliters of the bacterial suspensions were added to microtiter plates containing, in each well, two-fold diluted drugs in 100 µL of Middlebrook 7H9 medium with 10% OADC. The volume of drug-free and bacteria-free wells was adjusted to 200 µL and used as the control. The plates were incubated at 37 °C for 5 days. Twenty microliters of Alamar Blue dye (Accumed International, Westlake, OH, USA) and 12.5 µL of 20% sterilized Tween 80 reagent were added. The plates were re-incubated at 37 °C for 24 h, and the colors of all wells were recorded. The MIC was defined as the lowest drug concentration that prevented a color change from blue to pink.

### 2.4. Determination of the Killing Curve of ACA against M. tuberculosis

The time-kill curves were determined using the BACTEC MGIT 960 culturing system (Becton Dickinson) by a previously described method [17]. In brief, a log-phase culture of *M. tuberculosis* H37Rv was diluted with sterile water to reach a concentration equivalent to McFarland Standard No. 1 (approximately 3 × 10^7^ CFU/mL) and subsequently diluted 100-fold. Five hundred microliters of the diluted culture were inoculated into each MGIT 960 tube and incubated until the growth reached about 10,000 growth units (G.U.), when the bacterial growth was in the late log phase, which usually took 8–9 days. Three 100-µL aliquots of each incubated culture were transferred to three new tubes: one for a control and the others for testing with *rac*-ACA either at 5× MIC (13.5 µg/mL) or 1× MIC (2.7 µg/mL). The MGIT 960 tubes were further incubated, and at different time points (30, 60, 90, 120, 150 180, 210, 240, 270, 300 min, 1, 2, 3, 4, 5, 6 and 7 days), 100 μL of the culture were transferred to a new tube and incubated until the G.U. reached 4000–10,000. The number of days to positive (DTP) was recorded and used for calculation of CFU by comparison with the standard curve constructed by plotting the CFU versus the DTP of various dilutions of the bacteria grown in drug-free medium. The *M. tuberculosis* time-kill curves after exposure to either 5× MIC of RMP (2.0 µg/mL) or 5× MIC of INH (0.5 µg/mL) were done similar to positive controls and for preliminarily comparing the killing rate. The concentrations were used to make sure that all were well above the minimum bactericidal concentration (MBC). All assays were performed in duplicate.

## 3. Results

The MIC of ACA purified from *A. galanga* (*S*-ACA) against standard *M. tuberculosis* H37R*a* and H37R*v* strains was lower than that of the synthetic racemic ACA (*rac*-ACA), by 3–4-fold (Table 1). These results were confirmed repeatedly as both reference strains were included as controls when MICs against clinical isolates were determined.

The MICs of ACA against clinical isolates were subsequently determined. *S*-ACA was initially tested against 100-sensitive, 20 monodrug-resistant and 30 multidrug-resistant clinical *M. tuberculosis* isolates obtained from Ramathibodi Hospital. In total, 37%, 98% and 100% of the isolates were sensitive to *S*-ACA at 0.5, 1.0 and 2.0 μg/mL, respectively, and the sensitivity profiles of the drug-sensitive, monodrug-resistant and MDR isolates were not significantly different (Table 2). There was no correlation between the MIC of *S*-ACA and the resistance to any particular drugs (Table S1).

To further explore the sensitivity profile among MDR isolates, *rac*-ACA was tested against 93 additional drug-resistant isolates obtained from Siriraj Hospital, including one RMP-OFX-resistant, 40 MDR, 24 quinolone-resistant MDR (pre-XDR) and 28 XDR isolates. At 1.0 and 2.0 μg/mL, 55 and 98% of the isolates, respectively, were sensitive to *rac*-ACA. Two isolates exhibited higher MICs. A MDR isolate resistant to INH, RMP and EMB was inhibited by *rac*-ACA at 4.0 μg/mL, while another pre-XDR isolate resistant to INH, RMP, EMB, SM and OFX was inhibited at 16.0 μg/mL. The pre-XDR isolates had slightly, but significantly higher MICs to *rac*-ACA than the MDR isolates (chi-squared test, *p* = 0.0324) (Table 3). There was no correlation between the MICs of *rac*-ACA and any particular drugs (Table S2).

**Figure 2:**
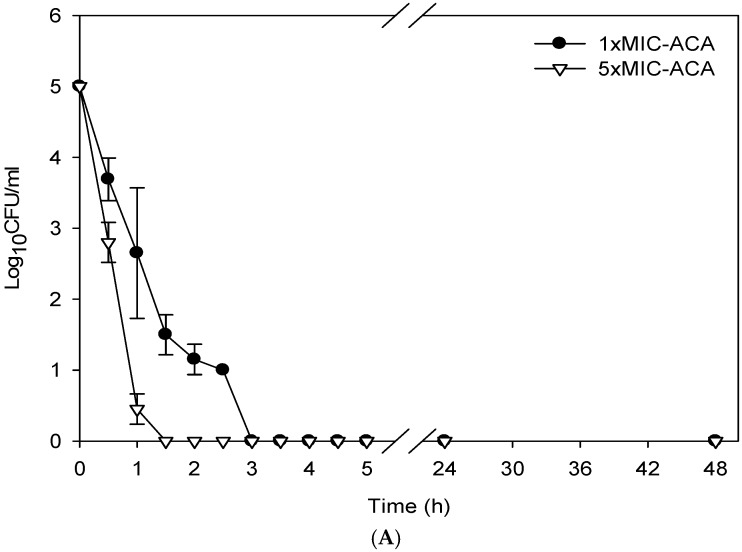

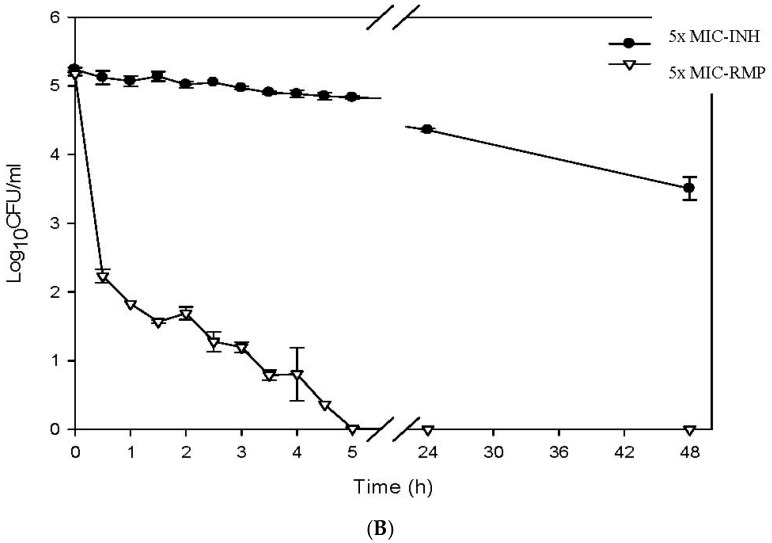
The time-kill curves of *M. tuberculosis* H37R*v* exposed to (**A**) *rac*-ACA at the concentrations of 13.5 µg/mL (5× MIC) and 2.7 µg/mL (1× MIC) and (**B**) 0.5 µg/mL of INH (5× MIC) and 2.0 µg/mL of RMP (5× MIC). Mean values and error bars of standard deviation values.

As a bactericidal drug would be highly valuable for treating tuberculosis, the bactericidal effect of ACA was tested, by determining the killing curve against the standard H37R*v* strain. As the bactericidal effect may be apparent at a higher concentration than MIC, the time-kill curves were investigated at the concentration of 1× MIC (2.7 µg/mL) and 5× MIC (13.5 µg/mL). The time-kill curves of RMP and INH at 5× MIC, 2 and 0.5 µg/mL respectively, were determined to compare the rate of killing. The time-kill curves of 0.4 µg/mL RMP and 0.1 µg/mL INH against the H37R*v* strain were also determined, but the killing effects were small in the same period of observation (data not shown). Exposure of *M. tuberculosis* H37R*v* to *rac*-ACA resulted in rapid and dramatic declines of viable cells (Figure 2A). At 5× MIC, 99% of bacterial cells were killed after an hour of exposure, and all were completely eliminated after 90 min. A similar trend was found when the bacilli were exposed to 1× MIC of *rac*-ACA. The mycobacterial cells were completely killed after 3 h of exposure. In contrast, *M. tuberculosis* survived up to 5 h after exposure to 5× MIC of RMP, whereas the killing by 5× MIC of INH was much slower. Only a small fraction was killed in the first 5 h (Figure 2B).

## 4. Discussion

As the incidence of MDR-TB is slowly increasing and the emergence of pre-XDR and XDR-TB has been reported, it is urgent to develop new anti-tuberculosis drugs. Although the anti-mycobacterial activity of the essential oils from *A. galanga* has long been recognized [18], few follow-up studies have been reported. Many demonstrated that ACA is a major constituent of the organic extract of *A. galanga*, although the exact amount might vary. Natural ACA has been purified from *A. galanga*, and its structure has been determined by chiral NMR, showing its *S*-configuration [13]. ACA itself has been shown to possess activity against the standard *M. tuberculosis* H37R*v* strain [5,6]. Here, we demonstrate that ACA maintains potent activity against the majority of a large number of clinical *M. tuberculosis* isolates, including MDR and XDR-TB isolates. It was also demonstrated that ACA had an interesting bactericidal activity. It was further demonstrated that the killing by *rac*-ACA was faster than either by INH or RMP at 5× MIC, although the absolute concentration of *rac*-ACA was higher. The killing curves of *rac*-ACA were studied in only the H37R*v* strain. Nevertheless, the results suggested that ACA and its derivatives are worthwhile leads for further study of targets and/or mechanisms.

The general use of *A. galanga* rhizome as a condiment for cooking suggests low general toxicity. It was shown that ACA was minimally toxic to several cell lines, demonstrated by using the neutral red assay to detect toxicity to rat intestinal epithelial cells (IEC6) after exposure up to 10 μM (2.34 μg/mL) [15]. Mokkhasmit demonstrated a high survival rate of mice after consumption of 100 mg of ACA per kg of body weight per day for 90 days [19]. It is interesting to note that even with the general human exposure to galangal in Thailand, *M. tuberculosis* resistance to ACA in nature is infrequent. As ACA contains two ester bonds, it is likely to be hydrolyzed in the mammalian gastrointestinal tract. Mori and his team [20] observed the instability of ACA during steam distillation. Moreover, Yang and Eilerman [12] reported that *S*-ACA easily undergoes hydrolysis and isomerization reactions in water or aqueous ethanol. After a few hours of storage at room temperature, three hydrolysis products were identified as: 1’-hydroxychavicol acetate, *p*-acetoxycinnamic alcohol and *p*-coumaryl diacetate. The rate of degradation increased with temperature and acidity. However, the reactions can be slowed at a lower temperature, in higher alcohol concentration and in a medium with a higher pH value. Collectively, it is reasonable to hypothesize that the majority of orally-ingested ACA is degraded in the gastrointestinal tract.

Because the allylic acetate structure facilitates the hydrolytic reactions, modification of the chemical structure could improve the stability [21]. Thus, while the possibility to develop ACA itself into an oral anti-TB drug may be remote, synthetic compounds that contain the ACA pharmacophore may be more likely to represent viable leads.

It has been determined previously that the chemical configuration of natural ACA was the *S*-form, which may be responsible for the activities of ACA [2,13,14,22]. It is unclear whether the *R*-form also possesses biological activity. As the *R*-form has not been found in nature, only natural *S*-ACA and *rac*-ACA were compared in this study. The MICs of *rac*-ACA against *M. tuberculosis* H37R*a* and H37R*v* were approximately 3–4-fold higher than those of *S*-ACA. This result suggests that the potent antimycobacterial activity is attributable primarily to the *S*-form. This configuration specificity implies that *S*-ACA might act on a specific molecular target in *M. tuberculosis*, despite its relatively simple molecular structure.

Both *S*- and *rac*-ACA were active against most clinical isolates of *M. tuberculosis* including MDR, pre-XDR and XDR mycobacterial clinical strains. This suggested that ACA does not act on the targets affected by currently-used anti-tuberculosis drugs. ACA had a potent bactericidal activity and was able to kill the H37R*v* strain completely after 3 h of exposure to 2.7 μg/mL. The killing effect seems to be superior to the first-line drugs, INH and RMP, at 5× MIC, although confirmation of the effects at various concentrations and in other strains is needed. Further studies on ACA, particularly on its killing kinetics, mode of action and molecular targets, are warranted.

## 5. Conclusions

The results of this study show that *S*-ACA acts on a specific target, is active against most clinical isolates and exhibits potent bactericidal activity. However, it may be metabolically unstable. This indicates that *S*-ACA potentially serves as a lead that could inspire the synthesis of more stable analogs and might lead to the identification of a specific target for TB drug development.

## Figures and Tables

**Table 1:** Minimum inhibitory concentrations (MICs) of 1′-*S*-acetoxychavicol acetate *(S*-ACA) purified from *A. galanga*, racemic ACA (*rac*-ACA) and rifampicin (RMP) against *M. tuberculosis* H37R*a* and H37R*v* strains.

Compound	MIC (µg/mL)	
	H37R*a* ATCC 25177	H37R*v* ATCC 27294
*S*-ACA	0.2	0.7
*rac*-ACA	0.5	2.7
RMP	0.005	0.1

**Table 2:** MICs of *S*-ACA against 150 clinical *M. tuberculosis* isolates obtained from Ramathibodi Hospital. MDR, multidrug-resistant.

Groups of Clinical Isolates	MIC Values of *S*-ACA (μg/mL)				Number of Clinical Isolates
	0.25	0.5	1.0	2.0	
Drug susceptible	4 (4%)	33 (33%)	62 (62%)	1 (1%)	100
Mono-resistant	2 (10%)	6 (30%)	11 (55%)	1 (5%)	20
MDR	2 (6.7%)	11 (36.7%)	16 (53.3%)	1 (3.3%)	30
Total	8 (5.3%)	50 (33.3%)	89 (59.3%)	3 (2%)	150

Mono-resistant = resistance to one drug, either RMP, or isoniazid (INH), or ethambutol (EMB), or streptomycin (SM).

**Table 3:** MICs of *rac*-ACA against drug-resistant clinical *M. tuberculosis* isolates obtained from Siriraj Hospital.

Groups of Clinical Isolates	MIC Values of *rac*-ACA (μg/mL)						Number of Clinical Isolates
	0.25	0.5	1.0	2.0	4.0	16	
RMP-OFX-resistant	0	0	0	1 (100%)	0	0	1
MDR	0	1 (2.5%)	25 (62.5%)	13 (32.5%)	1 (2.5%)	0	40
Pre-XDR	0	2 (8%)	7 (29%)	14 (58%)	0	1 (4%)	24
XDR	0	4 (14%)	12 (43%)	12 (43%)	0	0	28
Total	0	7 (8%)	44 (47%)	40 (43%)	1 (1%)	1 (1%)	93

RMP-OFX-resistant = resistance to RMP and ofloxacin (OFX); MDR = resistance to RMP and INH; Pre-extensively drug-resistant (XDR) = resistance to RMP, INH and OFX; XDR = resistance to RMP, INH and OFX and kanamycin (KAN).

## Supplementary Materials

The following are available online at www.mdpi.com/2218-0532/85/3/32/s1, Table S1: MICs of *S*-ACA for clinical isolates with different resistant profiles obtained from Ramathibodi Hospital; Table S2. MICs of *rac*-ACA (*R,S*-form) against drug-resistant clinical isolates with different resistant profiles obtained from Siriraj Hospital. Drug susceptibility testing was performed by the proportion method with the critical concentration of drugs as follows: RMP 1.0 μg/mL, INH 0.2 μg/mL, EMB 5.0 μg/mL, streptomycin (SM) 2.0 μg/mL, KAN 6.0 μg/mL and OFX 2.0 μg/mL.
