# Supplementary material for: In Vitro Activities of Enantiopure and Racemic 1′-Acetoxychavicol Acetate against Clinical Isolates of Mycobacterium tuberculosis
Source: Sci Pharm. 2017 Sep 18;85(3):32. doi: 10.3390/scipharm85030032 (PMC5620519; doi:10.3390/scipharm85030032)
Supplement: Supplementary file 1 [file scipharm-85-00032-s001.pdf]

Supplementary Material

In Vitro Activities of Enantiopure and Racemic 1′-Acetoxychavicol Acetate against Clinical Isolates of *Mycobacterium tuberculosis*

Table S1. Minimum inhibitory concentrations of 1′-*S*-acetoxychavicol acetate (*S*-ACA) for clinical isolates with different resistant profiles obtained from Ramathibodi Hospital.

Resistant to:	MICs of *S*-ACA (μg/mL)				Number of Clinical Isolates
	0.25	0.5	1.0	2.0	
INH	-	-	4	-	4
RMP	-	-	2	-	2
EMB	-	-	1	-	1
SM	1	5	2	1	9
INH, SM	-	1	1	-	2
RMP, EMB	-	-	1	-	1
INH, EMB, SM	1	-	-	-	1
INH, RMP	-	2	4	1	7
INH, RMP, EMB	-	2	1	-	3
INH, RMP, SM	1	4	8	-	13
INH, RMP, EMB, SM	1	3	3	-	7
Total	4	17	27	2	50

INH: isoniazid; RMP: rifampicin; EMB: ethambutol; SM: streptomycin.

Table S2. MICs of *rac*-ACA (*R*,*S*-form) against drug-resistant clinical isolates with different resistant profiles obtained from Siriraj Hospital. Drug susceptibility testing was performed by the proportion method with the critical concentration of drugs as follows: RMP 1.0 μg/mL, INH 0.2 μg/mL, EMB 5.0 μg/mL, SM 2.0 μg/mL, KAN 6.0 μg/mL and OFX 2.0 μg/mL.

**Resistant to**	**MICs of *rac*-ACA (μg/mL)**						**Number of Clinical Isolates**
	0.25	0.5	1.0	2.0	4.0	16.0	
RMP, OFX	-	-	-	1	-	-	1
INH, RMP	-	1	8	4	-	-	13
INH, RMP, EMB	-	-	5	2	1	-	8
INH, RMP, SM	-	-	5	3	-	-	8
INH, RMP, EMB, SM	-	-	7	4	-	-	11
INH, RMP, OFX	-	1	1	1	-	-	3
INH, RMP, EMB, OFX	-	-	-	2	-	-	2
INH, RMP, SM, OFX	-	-	1	1	-	-	2
INH, RMP, EMB, SM, OFX	-	1	5	10	-	1	17
INH, RMP, KAN, OFX	-	-	1	1	-	-	2
INH, RMP, EMB, KAN, OFX	-	2	5	1	-	-	8
INH, RMP, SM, KAN, OFX	-	-	2	6	-	-	8
INH, RMP, EMB, SM, KAN, OFX	-	2	4	4	-	-	10
**Total**	-	7	44	41	1	1	93

INH: isoniazid; RMP: rifampicin; EMB: ethambutol; SM: streptomycin., OFX: ofloxacin; KAN: kanamycin
